# Supplementary material for: Sucrose-Based Screening of a Novel Strain, Limimaricola sp. YI8, and Its Application to Polyhydroxybutyrate Production from Molasses
Source: Polymers (Basel). 2025 May 26;17(11):1471. doi: 10.3390/polym17111471 (PMC12158085; doi:10.3390/polym17111471)
Supplement: Supplementary file 1 [file polymers-17-01471-s001.zip › polymers-3615866-supplementary.pdf]

Supplementary Material

Supplementary Figure

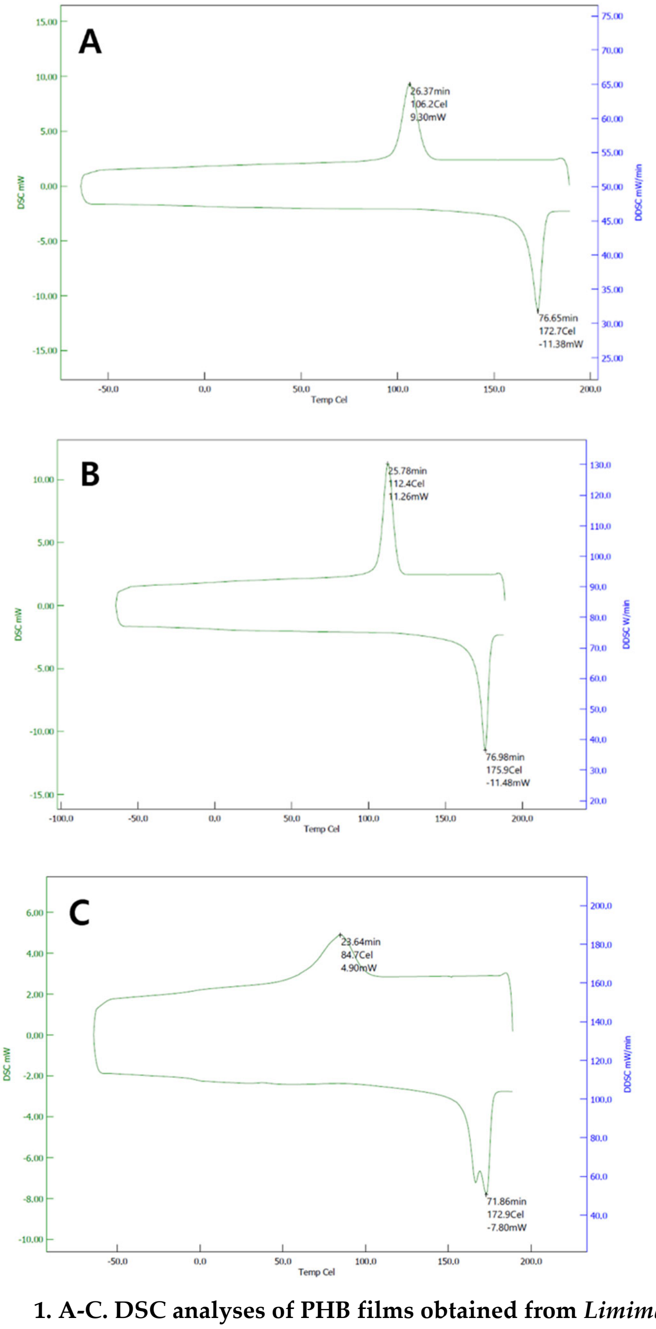

1. A-C. DSC analyses of PHB films obtained from *Limimaricola* sp. YI8, *Escherichia coli*, and *Cupriavidus necator* H16. (A) DSC analysis of the YI8-PHB film, (B) DSC analysis of the *E. coli*-PHB film, and (C) DSC analysis of the *C. necator* H16-PHB film.

Supplementary Tables

Table S1. High(+1) and low(-1) values of independent variables in the Plackett-Burman design

Factor	Levels of factor	
	-1	1
Peptone (X_1, g/L)	2.5	10
Yeast extract (X_2, g/L)	0.5	2
Sodium Chloride (X_3, g/L)	10	30
Sucrose (X_4, g/L)	5	20
$MgCl_2$ (X_5, g/L)	1.18	5.9
$MgSO_4$ (X_6, g/L)	0.648	3.24
CaCl2 (X_7, g/L)	0.36	1.8
K_2SO_4 (X_8, g/L)	0.11	0.55
$NaHCO_3$ (X_9, g/L)	0.016	0.16
Boric acid (X_{10}, mg/L)	2.2	22
Dummy1 (D_1, g/L)	-1	1
Dummy2 (D_2, g/L)	-1	1

Table S2. Combinations of variables and responses in the Plackett–Burman design experiment

Run	Independent variables										Dummy variables	
	X1	X2	X3	X4	X5	X6	X7	X8	X9	X10	D1	D2
1	-1	1	-1	1	-1	1	1	1	1	-1	-1	1
2	1	-1	-1	-1	-1	1	-1	1	-1	1	1	1
3	1	-1	1	1	-1	-1	-1	-1	1	-1	1	-1
4	-1	1	1	1	1	-1	-1	1	1	-1	1	1
5	1	-1	1	-1	1	1	1	1	-1	-1	1	1
6	-1	1	1	-1	1	1	-1	-1	-1	-1	1	-1
7	-1	1	1	-1	-1	-1	-1	1	-1	1	-1	1
8	-1	-1	-1	-1	1	-1	1	-1	1	1	1	1
9	1	1	-1	-1	1	1	-1	1	1	-1	-1	-1
10	-1	1	-1	1	1	1	1	-1	-1	1	1	-1
11	1	1	1	1	-1	-1	1	1	-1	1	1	-1
12	-1	-1	-1	-1	-1	-1	-1	-1	-1	-1	-1	-1
13	1	1	-1	1	1	-1	-1	-1	-1	1	-1	1
14	-1	-1	-1	1	-1	1	-1	1	1	1	1	-1
15	1	1	-1	-1	-1	-1	1	-1	1	-1	1	1
16	-1	-1	1	1	-1	1	1	-1	-1	-1	-1	1
17	-1	-1	1	-1	1	-1	1	1	1	1	-1	-1
18	1	1	1	-1	-1	1	1	-1	1	1	-1	-1
19	1	-1	-1	1	1	-1	1	1	-1	-1	-1	-1
20	1	-1	1	1	1	1	-1	-1	1	1	-1	1

Table S3. Values of Factors in the Box-Behnken design

Factor	Levels of factor		
	-1	0	1
Yeast extract (X_2, g/L)	0	1	2
NaCl (X_3, g/L)	0	15	30
Sucrose (X_4, g/L)	5	30	50

Table S4. Box-Behnken design matrix with experimental values of cell mass and PHB production

Run	NaCl	Sucrose	Yeast extract	DCW (g/L)	PHB (g/L)
1	0	0	0	1.91	0.38
2	0	0	0	2.08	0.42
3	1	0	-1	0	0
4	0	0	0	2.21	0.44
5	-1	1	0	2.60	0.52
6	-1	0	-1	0	0
7	0	-1	1	1.31	0.26
8	-1	0	1	1.81	0.36
9	1	0	1	0.15	0.03
10	0	-1	-1	0.25	0.05
11	0	1	-1	0	0
12	0	1	1	1.94	0.39
13	-1	-1	0	0.81	0.16
14	1	1	0	0.04	0.01
15	1	-1	0	0.23	0.05
